# STNet: A novel spiking neural network combining its own time signal with the spatial signal of an artificial neural network

**DOI:** 10.3389/fnins.2023.1151949

**Published:** 2023-04-18

**Authors:** Fang Liu, Wentao Tao, Jie Yang, Wei Wu, Jian Wang

**Affiliations:** ^1^School of Mathematical Sciences, Dalian University of Technology, Dalian, China; ^2^Key Laboratory for Computational Mathematics and Data Intelligence of Liaoning Province, Dalian, China; ^3^College of Science, China University of Petroleum (East China), Qingdao, China

**Keywords:** hybrid network, spiking neural network, artificial neural network, spatio-temporal information, STNet

## Abstract

**Introduction:**

This article proposes a novel hybrid network that combines the temporal signal of a spiking neural network (SNN) with the spatial signal of an artificial neural network (ANN), namely the Spatio-Temporal Combined Network (STNet).

**Methods:**

Inspired by the way the visual cortex in the human brain processes visual information, two versions of STNet are designed: a concatenated one (C-STNet) and a parallel one (P-STNet). In the C-STNet, the ANN, simulating the primary visual cortex, extracts the simple spatial information of objects first, and then the obtained spatial information is encoded as spiking time signals for transmission to the rear SNN which simulates the extrastriate visual cortex to process and classify the spikes. With the view that information from the primary visual cortex reaches the extrastriate visual cortex *via* ventral and dorsal streams, in P-STNet, the parallel combination of the ANN and the SNN is employed to extract the original spatio-temporal information from samples, and the extracted information is transferred to a posterior SNN for classification.

**Results:**

The experimental results of the two STNets obtained on six small and two large benchmark datasets were compared with eight commonly used approaches, demonstrating that the two STNets can achieve improved performance in terms of accuracy, generalization, stability, and convergence.

**Discussion:**

These prove that the idea of combining ANN and SNN is feasible and can greatly improve the performance of SNN.

## 1. Introduction

At present, artificial neural networks (ANNs), especially deep neural networks, have become the tool of choice for many machine learning tasks. They have been successfully applied to many fields such as pattern recognition (Abiodun et al., 2019), automatic control (Seo, 2013), biology (Kang et al., 2017), medicine (Huang et al., 2019), as well as economics (Li and Ma, 2010), and achieved exciting results. The theoretical research of ANNs has also achieved fruitful results, reflected in structural adjustment, selection of activation functions, improvement of learning methods, etc. The applications of other techniques such as weight initialization, batch normalization, regularization, and dropout also enable ANNs to achieve advanced performance and strong generalization.

However, there is a fatal flaw in ANNs. The internal state is calculated and expressed by continuous signals in the spatial domain (Xie et al., 2020). This makes ANNs not only farther from real human brain activity (Cheng et al., 2020), but also increases energy consumption and processing demands at an unsustainable speed for higher accuracy (Davidson and Furber, 2021), which further limits the potential of neuromorphic hardware.

In contrast, a spiking neural network (SNN) uses discrete spiking signals belonging to the time domain, instead of the continuous values in an ANN, to process and transmit information (Xie et al., 2020). Each dynamic spiking neuron fires a spike when the internal state of the neuron (i.e., the membrane potential) reaches a certain threshold. Evidence reveals that in the biological neuron, actual film channels control the movements of particles over the layer by opening and shutting in light of voltage changes because of inborn current flows and remotely led to signals (Rafi, 2021). The human brain disposes of information between neurons through electrical motivations (Taherkhani et al., 2020). Therefore, due to its discrete spiking signals and dynamics, an SNN is more biologically realistic and biologically interpretable than an ANN (Fu and Dong, 2022). Meanwhile, because of its discrete and efficient event-driven computing, an SNN consumes less energy than an ANN in the implementation of neuromorphic hardware (Kheradpisheh and Masquelier, 2020).

However, the performance of spiking neural networks is not yet satisfactory (Muramatsu and Yu, 2021). There are several reasons for this. First, the non-differentiable property of the spike activity makes the excellent gradient descent algorithm inapplicable in SNN (Hao et al., 2020). Second, due to the discrete nature of the spiking mechanism, the continuous-value dataset is forced to be converted into a spiking time series before being fed into the SNN, which may lead to information loss and further adversely affect the SNN experimental results (Muramatsu and Yu, 2021).

There are currently two ways to solve these problems. One is to improve the SNN by fully mimicking biological realities. For example, spike-timing dependent plasticity (STDP) observed in mammalian visual cortex (Kheradpisheh et al., 2018) is usually used to update synaptic weights locally based on pre- and post-spike activities. It is then developed into a supervised learning rule, such as dopamine-modulated STDP, which has been observed in several different experiments on the hippocampus and prefrontal cortex (Nobukawa et al., 2019). In order to simulate the random synaptic connections of the biological network in the human brain, Zhao et al. (2021) applies Dropout and DropConnect technologies in the SNN. Nonetheless, it is still not possible to make an SNN reach the performance of an ANN through only analogous biological realities.

The other way of thinking is to improve SNN by drawing on the advanced ANN. For instance, the ANN-SNN conversion scheme, that is, copying the weights of a trained ANN to an SNN with the same structure, is a common training method (Rueckauer et al., 2017), but it requires a large number of time steps and brings accuracy loss during the conversion process. The surrogate gradient method (Stewart and Neftci, 2022) is a gradient descent method that works for SNNs with some approximation assumptions. However, finding differentiable alternatives to neuron functions to match the performance of ANNs remains a challenge (Nguyen et al., 2021). Zhang et al. (2021) proposed the Rectified Linear Postsynaptic Potential function as a new spiking neuron model by analogy with the Rectified Linear Unit (ReLU) function in ANN and alleviates dead neuron problem.

In addition, the direct combination of SNNs and ANNs has been verified as an effective choice to improve the performance of SNNs, because it can give full play to the advantages of both. Xu et al. (2018) proposed a convolutional neural network (CNN)-SNN model to improve the feature extraction ability of SNNs by using CNN extracting image features for SNN. However, its CNN and SNN parts are trained separately, which increases the computational burden. Muramatsu and Yu (2021) built a versatile hybrid neural network by combining an ANN and an SNN, and its accuracy is verified to be close to that of ANN but it ignores the combination of temporal and spatial information.

The human brain is a complex and comprehensive spatio-temporal information processing machine (Kasabov, 2014). Spatio-temporal information in biological neural systems enables the human brain to work efficiently with high-density information representations because not only space but also time carry information (He et al., 2019). Therefore, in this article, we propose a combined network, namely the Spatio-Temporal Combined Network (STNet). Different from existing networks, the STNet pays more attention to the combination of the spatial signal extracted from the ANN and the spiking time signal from the SNN. This makes up for the defect that an ANN or an SNN only have a single signal to express information, thereby increasing the richness of information to develop advantages from both. Moreover, the STNet realizes simultaneous learning in the ANN and SNN layers *via* the joint use of the gradient descent and SpikeProp method (Bohte et al., 2002) instead of training the SNN part after completing the ANN training.

The originality of this article is that we provide two types of STNet, a concatenated version, C-STNet, and a parallel version, P-STNet. The construction of these two architectures is motivated by the simulation of the processing of visual information in the visual cortex. Visual information is sent to the extrastriate visual cortex for final visual processing by the primary visual cortex (Joukal, 2017). Inspired by this process, in the C-STNet, we use an ANN to extract the simple spatial information of the datasets, and convert its output to spike times as the inputs of an SNN. Then, the SNN is regarded as the extrastriate visual cortex for classification. Furthermore, in the view that information from the primary visual cortex reaches the extrastriate visual cortex *via* the ventral and dorsal streams (Joukal, 2017), for the P-STNet, we combine an ANN and an SNN in parallel to jointly extract the spatio-temporal information of objects, and transmit the processed spiking time to an SNN for classification. Notice that the processed spiking time is the combination of the initial SNN's outputs and the converted counterparts from the spatial signals extracted from the ANN.

The rest of the article is organized as follows. The preliminary knowledge about the neuron model of an ANN and an SNN is introduced in Section 2. The structures and learning algorithms of the C-STNet and the P-STNet are given in Section 3. The classification experiments to evaluate the C-STNet and the P-STNet are presented in Section 4. Conclusions are provided in Section 5.

## 2. Preliminary knowledge

### 2.1. Neuron model of ANN

A fully connected ANN is made up of multiple layers of neurons. The neurons in the previous layer transmit values forward, while the posterior neuron adds all these weighted inputs and uses the activation function to map this summation. Therefore, the output of the posterior neuron can be written as


(1) $$\begin{array}{l} R_{q}=f\left(\Sigma_{n\in \Gamma_{q}}w_{nq}x_{n}\right), \end{array}$$

Where Γ_*q*_ denotes the set of all previous neurons connected to the posterior neuron *q*. *w*_*nq*_ is the weight between the previous neuron *n* and the posterior neuron *q*. *x*_*n*_ denotes the output of the previous neuron *n*. *f*(·) is the activation function, and we use the sigmoid function in this article.

### 2.2. Neuron model of SNN

**Figure 1:**
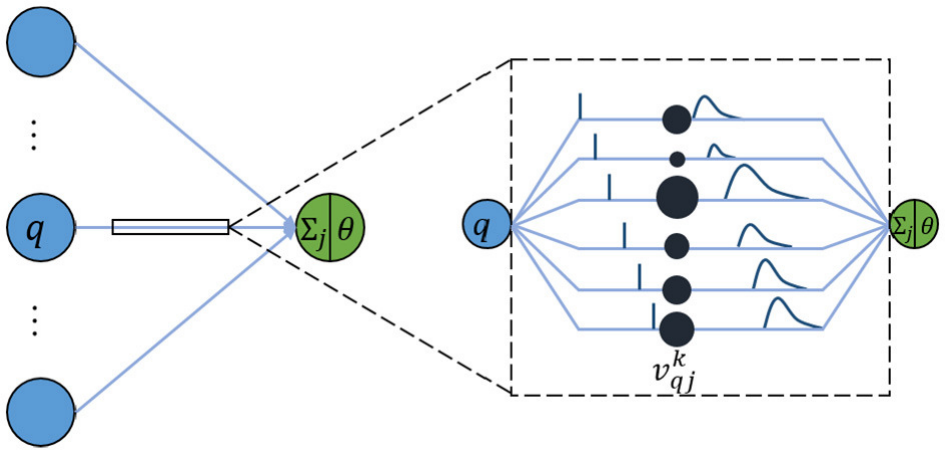
Schematic diagram of SRM.

In a like manner, an SNN is composed of multiple-layer spiking neurons. For the basic unit, the spike response model (SRM) is one of the commonly used neuron models because it conforms to biological reality and requires few calculations (Amin et al., 2017). Although it looks similar to the neuron model of an ANN, each connection between two neurons contains multiple synapses as shown in Figure 1. For simplicity, it is assumed that each neuron generates at most one spike during the simulation time window interval *T*. The details of the specific calculation are described as follows.

In an SRM, each presynaptic neuron transmits the spiking time to the postsynaptic neuron through *K* synapses, where each synapsis has a different time delay. The postsynaptic neuron *j* receives a set of spikes from all its presynaptic neurons to enhance its internal state variable, namely membrane potential *u*_*j*_(*t*), and will fire a spike if *u*_*j*_(*t*) crosses a threshold θ. The moment when *u*_*j*_(*t*) reaches θ is called the firing time. Therefore, the firing time *t*_*j*_ is a nonlinear function of *u*_*j*_:


(2) $$\begin{array}{l} t_{j}=t_{j}\left(u_{j}\right). \end{array}$$

The membrane potential *u*_*j*_(*t*) of neuron *j* is defined as the sum of all weighted post-synaptic potentials (PSPs) produced by the received spiking time series.


(3) $$\begin{array}{l} u_{j}\left(t\right)=\Sigma_{q\in \Sigma_{j}}\Sigma_{k=1}^{K}v_{qj}^{k}y_{q}^{k}\left(t\right), \end{array}$$

Where Γ_*j*_ denotes the set of all presynaptic neurons connected to the postsynaptic neuron *j*. $v_{qj}^{k}$ represents the weight of the *k*-th synapse between the neuron *q* in the previous layer and the postsynaptic neuron *j*. $y_{q}^{k}\left(t\right)$ denotes the PSP, given as


(4) $$\begin{array}{l} y_{q}^{k}\left(t\right)=\epsilon \left(t-t_{q}-d^{k}\right), \end{array}$$

Where *t*_*q*_ is the spiking time fired by the presynaptic neuron *q*, and *d*^*k*^ is the time delay associated with synaptic terminal *k*. ϵ(*s*) is the spike response kernel employed to simulate the PSP defined as


(5) $$\begin{array}{l} \epsilon \left(s\right)=\{\begin{array}{cc} \frac{s}{\tau }\exp\left(1-\frac{s}{\tau }\right), & s>0\\ 0, & s\leq 0 \end{array}, \end{array}$$

Where τ is a time constant to determine the shape of ϵ(*s*). If the membrane potential of the postsynaptic neuron does not reach the threshold within the time window limitation, the neuron will not emit any spikes. For simplicity, it is stipulated that a neuron fires one spike at most.

## 3. Methods

In this section, we introduce a novel kind of spiking neural network, namely the Spatio-Temporal Combined Network (STNet), which combines the spatial domain information delivered by an ANN and the time domain information transmitted by an SNN. Two versions are described separately below, a concatenated version named C-STNet and a parallel version named P-STNet.

### 3.1. Network structure of the C-STNet

**Figure 2:**
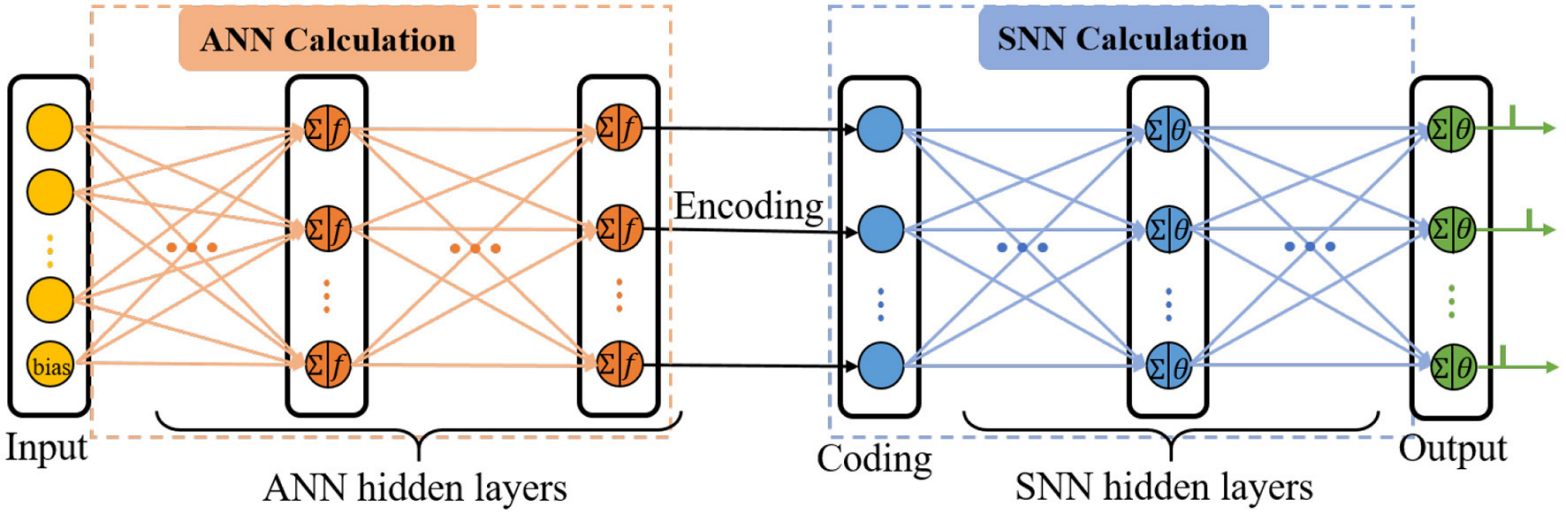
The topology structure of the C-STNet.

C-STNet is the concatenated version to realize the serial combination of spatio-temporal information by stacking an ANN on the front and an SNN on the back, as shown in Figure 2. The operating mechanism of the former module is a simple ANN, while the latter module is an ordinary SNN. They are connected by a coding operation that converts the spatial information extracted from the ANN into temporal information.

The input layer of the ANN is responsible for receiving data and feeding it into the C-STNet, where each neuron represents a feature of the data, and an additional bias neuron is added. Then, several ANN hidden layers follow and the neurons of which perform the ANN neuron model operations described in Section 2.1. The bias neuron of the input layer is connected in pairs with all neurons of the adjacent ANN hidden layer like other feature neurons.

The coding layer is indispensable for realizing the serial conversion of spatio-temporal information. Its neurons are in one-to-one correspondence with the neurons in the last layer of the ANN's hidden layers. Considering that the higher the spatial feature value extracted by the ANN corresponds to the earlier the spike in the SNN is fired, the coding operation is defined as the reciprocal form


(6) $$\begin{array}{l} t_{q}=\frac{\beta }{R_{q}}, \end{array}$$

Where *R*_*q*_ is the output of the *q*-th neuron in the last layer of the ANN hidden layers. β is a constant parameter and is not learnable. *t*_*q*_ is the output of the coding neuron *q*. If *t*_*q*_ exceeds the time interval, i.e., *t*_*q*_>*T*, *t*_*q*_ is forcibly set to *T*.

Next, the spiking time is transmitted in the manner of the SRM neuron model as described in Section 2.2. When the membrane potential of the output neuron crosses the threshold, it will emit a spike. Finally, the C-STNet classifies the input sample into the category corresponding to the output neuron of the first triggered spike.

### 3.2. Network structure of the P-STNet

**Figure 3:**
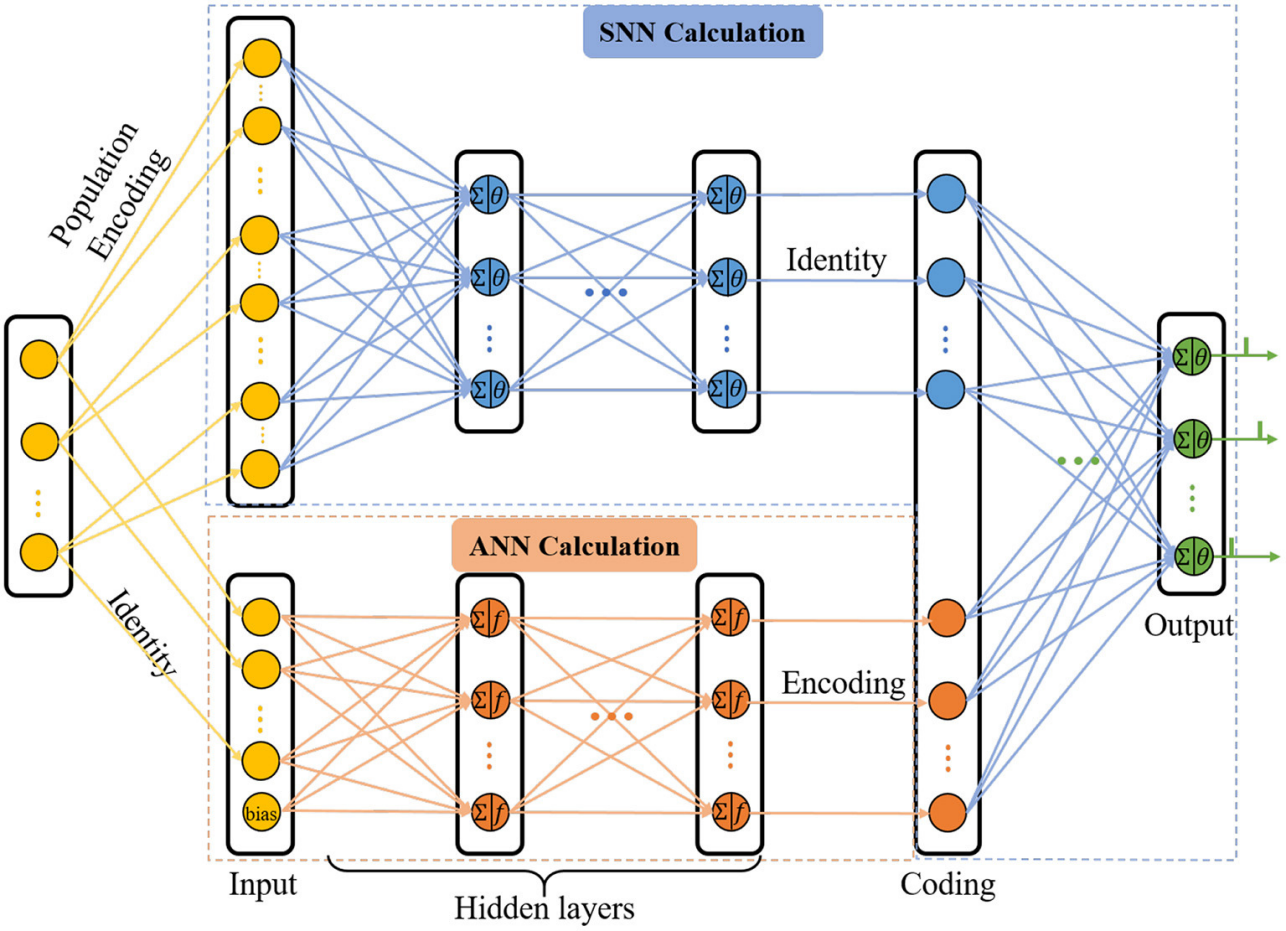
The topology structure of P-STNet.

With the view that the human brain is a complex integrated spatio-temporal system, it is thus essential for methodologies to operate over both the space and time domains (Wang et al., 2018). In order to extract the spatio-temporal information of the network, we updated the C-STNet to a dual-path structure, namely the P-STNet, as shown in Figure 3. Specifically, P-STNet first divides into two paths, like the biological ventral and dorsal streams, using the raw data for parallel calculations in an SNN and an ANN and then combines the two paths in the form of serial splicing in the coding layer. Finally, the P-STnet executes classification through the rear SNN. The calculation details of the newly added upper path SNN and the rear SNN are the same as the SNN part in the C-STNet, except that the number of neurons contained in each layer may be different. The operation scheme of its lower path is the same as the corresponding part in the C-STNet.

It is worth mentioning that before the raw dataset is fed into the upper path, it needs to be encoded into the spiking trains. A popular population coding scheme that is usually used is given in Figure 3, which is a good choice for promoting an SNN to higher performance (Pan et al., 2019). Specifically, each feature of the sample is converted to G spikes by *G* Gaussian fields. Therefore, the number of input neurons in the upper path is equal to *N*×*G*, where *N* is the number of the features.

In the cases where a dataset contains too many features, adopting the population coding scheme will generate a large number of input neurons, thus increasing the computational burden. At this time, we adopted a simpler linear time delay coding as follows:


(7) $$\begin{array}{l} {\text{x}}^{c}=\frac{{\text{x}}_{\max}-\text{x}}{{\text{x}}_{\max}-{\text{x}}_{\min}}\times T, \end{array}$$

Where **x**∈ℝ^*N*^ is any sample, and **x**^*c*^ is its encoded counterpart. **x**_max_ and **x**_min_ represent the maximum and minimum values of each feature among all samples, respectively. The coding method follows the rule that large values correspond to early spikes and vice versa.

### 3.3. Learning algorithms

In this section, we use SpikeProp and gradient descent methods to derive the learning formulas of the weights in the two STNets and give their learning algorithms. Let (**x**, *y*) be any sample of the training dataset, where **x** = (*x*_1_, *x*_2_, ⋯ , *x*_*N*_) is an *N*-dimensional normalized feature vector. *y*∈ℤ is the category label of the sample, which is encoded as a series of expected trigger time ${\left\{t_{j}^{d}\right\}}_{j=1}^{J}$. *J* is the number of neurons in the output layer of STNet. Assuming ${\left\{t_{j}\right\}}_{j=1}^{J}$ is the corresponding actual trigger time of the STNet, the loss function of the network is


(8) $$\begin{array}{l} E\left(\text{W}\right)=\frac{1}{2}\Sigma_{j=1}^{J}{\left(t_{j}-t_{j}^{d}\right)}^{2}, \end{array}$$

Where $t_{j}^{d}$ and *t*_*j*_ are the *j*-th expected and actual network output spiking time, respectively. **W** is a combination of all weight parameters used in STNet.

For convenience, we tested a four-layer C-STNet (including an input layer, an ANN hidden layer, a coding layer, and an output layer) and a four-layer P-STNet (including an input layer, a hidden layer, a coding layer, and an output layer) as examples. For the C-STNet, during backpropagation from the output layer to the coding layer, the SpikeProp algorithm is executed to update the weights between these two layers. Given the weight $v_{qj}^{k}$, denoting the weight of the *k*-th synapse between the *q*-th neuron in the coding layer and the *j*-th neuron in the output layer, it is updated according to Equations (2)–(5) and (8) as


(9) $$\begin{array}{l} v_{qj}^{k}=v_{qj}^{k}+\Delta v_{qj}^{k}, \end{array}$$

and


(10) $$\begin{array}{l} \begin{array}{l} \Delta v_{qj}^{k}=-\eta \frac{\partial E\left(\text{W}\right)}{\partial v_{qj}^{k}}=-\eta \frac{\partial E\left(\text{W}\right)}{\partial t_{j}}\frac{\partial t_{j}}{\partial u_{j}\left(t_{j}\right)}\frac{\partial u_{j}\left(t_{j}\right)}{\partial v_{qj}^{k}}\\ =-\eta \left(t_{j}-t_{j}^{d}\right)\frac{-1}{\partial u_{j}\left(t\right)/\partial t\left(t_{j}\right)}y_{q}^{k}\left(t_{j}\right)\\ =-\eta \frac{t_{j}^{d}-t_{j}}{\Sigma_{q^{\prime }=1}^{Q}\Sigma_{k=1}^{K}v_{q^{\prime }}^{k}\left(t\right)\partial y_{q^{\prime }}^{k}\left(t\right)/\partial t\left(t_{j}\right)}y_{q}^{k}\left(t_{j}\right)\\ ≜-\eta \delta_{j}y_{q}^{k}\left(t_{j}\right), \end{array} \end{array}$$

where


(11) $$\begin{array}{l} \frac{\partial y_{q^{\prime }}^{k}\left(t\right)}{\partial t}\left(t_{j}\right)=\frac{1}{\tau }e^{1-\left(t_{j}-t_{q}-d^{k}\right)/\tau }-\frac{1}{\tau }y_{q^{\prime }}^{k}\left(t_{j}\right), \end{array}$$

and η∈(0, 1) is learning rate.

Then, the changes in the weights between the input layer and the ANN hidden layer are defined as


(12) $$\begin{array}{l} \Delta w_{nq}=-\eta \frac{\partial E\left(\text{W}\right)}{\partial w_{nq}}=-\eta \frac{\partial E\left(\text{W}\right)}{\partial t_{q}}\frac{\partial t_{q}}{\partial w_{nq}}, \end{array}$$

Where *w*_*nq*_ represents the weight between the *n*-th neuron in the input layer and the *q*-th neuron in the ANN hidden layer. $\frac{\partial E\left(\text{W}\right)}{\partial t_{q}}$ can be calculated as


(13) $$\begin{array}{l} \begin{array}{l} \frac{\partial E\left(\text{W}\right)}{\partial t_{q}}=\Sigma_{j=1}^{J}\delta_{j}\frac{\partial u_{j}\left(t_{j}\right)}{\partial t_{q}}\\ =\Sigma_{j=1}^{J}\delta_{j}\Sigma_{k=1}^{K}v_{qj}^{k}\partial y_{q}^{k}\left(t_{j}\right)/\partial t_{q}\\ =\Sigma_{j=1}^{J}\delta_{j}\Sigma_{k=1}^{K}v_{qj}^{k}\left(-\frac{1}{\tau }e^{1-\left(t_{j}-t_{q}-d^{k}\right)/\tau }+\frac{1}{\tau }y_{q}^{k}\left(t_{j}\right)\right) \end{array} \end{array}$$

According to Equations (1), (6), $\frac{\partial t_{q}}{\partial w_{nq}}$ is computed by


(14) $$\begin{array}{l} \frac{\partial t_{q}}{\partial w_{nq}}=\frac{\partial t_{q}}{\partial R_{q}}\frac{\partial R_{q}}{\partial w_{nq}}=\frac{-\beta }{{\left(R_{q}\right)}^{2}}R_{q}\left(1-R_{q}\right)x_{n}. \end{array}$$

Therefore, by synthesizing (Equations 12–14), Δ*w*_*nq*_ is expressed as


(15) $$\begin{array}{l} \begin{array}{l} \Delta w_{nq}=-\eta \left(\Sigma_{j=1}^{J}\delta_{j}\Sigma_{k=1}^{K}v_{qj}^{k}\frac{\partial y_{q}^{k}\left(t_{j}\right)}{\partial t_{q}}\right)\frac{-\beta }{{\left(R_{q}\right)}^{2}}R_{q}\left(1-R_{q}\right)x_{n}\\ =\eta \beta \left(\Sigma_{j=1}^{J}\delta_{j}\Sigma_{k=1}^{K}v_{qj}^{k}\frac{\partial y_{q}^{k}\left(t_{j}\right)}{\partial t_{q}}\right)\frac{R_{q}\left(1-R_{q}\right)x_{n}}{{\left(R_{q}\right)}^{2}}. \end{array} \end{array}$$

The weight adaptation rule is


(16) $$\begin{array}{l} w_{nq}=w_{nq}+\Delta w_{nq}. \end{array}$$

In summary, the pseudocode for training the C-STNet is given in Algorithm 1.

**Algorithm 1:** C-STNet.

**Input**: A training dataset.
// **Pretreatment**:
Normalize the features of each sample to (*x*_1_, *x*_2_, ⋯ , *x*_*N*_) by the maximum-minimum
mechanism;
Obtain every network input (*x*_1_, *x*_2_, ⋯ , *x*_*N*+1_) by adding the bias *x*_*N*+1_;
Encode the label of each sample to an expected spiking time series $\left(t_{1}^{d},t_{2}^{d},\cdots \;,t_{J}^{d}\right)$.
// **Initialization and settings**:
Initialize all parameters: **W**;
Initialize the epoch: *epoch* = 1;
Set the time constant τ of the spike response kernel and the spike interval limit *T*;
Set the learning rate η;
Set the number of hidden neurons and synapses, respectively;
Set the number of stop epochs: *Max*_*Epoch*;
Set the desired minimum of MSE: *Min*_*MSE*.
// **Training**:
**While** *epoch*<*Max*_*Epoch* and *MSE*>*Min*_*MSE* do
**For** every sample do
// **Forward propagation**:
Obtain the network outputs from according to (1), (6) and (2)-(5) in sequence.
// **Backward propagation**:
Update **W** using (9)-(16).
**End**
*epoch* = *epoch*+1.
**End**
Output: The optimal parameters W^*^.
Here the mean squared error (MSE) is the average of the sum of squared errors between the target outputs and corresponding outputs of the network for all samples.

For the P-STNet, the weight update rules between the output layer to the coding layer are the same as Equations (9)–(11), and the rules for the lower path are the same as Equations (12)–(16). Whereas, the weights **b** between the input layer and the hidden layer in the upper path are changed according to


(17) $$\begin{array}{l} b_{mp}^{k}=b_{mp}^{k}+\Delta b_{mp}^{k}, \end{array}$$

and


(18) $$\begin{array}{l} \Delta b_{mp}^{k}=-\eta \frac{\partial E\left(\text{W}\right)}{\partial b_{mp}^{k}}=\frac{\eta \left[\Sigma_{j=1}^{J}\delta_{j}\left(\Sigma_{k=1}^{K}v_{pj}^{k}\frac{\partial y_{p}^{k}\left(t_{j}\right)}{\partial t_{p}}\right)\right]y_{m}^{k}\left(t_{p}\right)}{\Sigma_{m=1}^{M}\Sigma_{k=1}^{K}b_{mp}^{k}\partial y_{m}^{k}\left(t\right)/\partial t\left(t_{m}\right)}, \end{array}$$

Where $b_{mp}^{k}$ is the weight between the *k*-th synapse of the presynaptic neuron *m* and the posterior neuron *p*. δ_*j*_ can be got through Equation (10). Therefore, the learning algorithm of P-STNet can be summarized in Algorithm 2, which is the same as Algorithm 1, except that during backward propagation Algorithm 2 updates weights using Equations (9)–(18). For simplicity, the flow scheme of Algorithm 2 is omitted here.

Notice that the learning rates in Algorithms 1, 2 are fixed. In order to progressively explore the optimal parameters on a finer scale as the training progresses, the learning rate can also be attenuated at a decay rate in each epoch.

## 4. Results

To evaluate the performance of the C-STNet and the P-STNet, we conducted experiments using six small datasets and two large datasets. In this section, we compare the experimental results of these two STNets with other eight existing approaches including seven SNNs: SpikeProp (Bohte et al., 2002), SWAT (Wade et al., 2010), SRESN (Dora et al., 2016), TMM-SNN (Dora et al., 2018), GE-SNN (López-Vázquez et al., 2019), SPDO (SpikeProp with Dropout) (Zhao et al., 2021), and SPDC (SpikeProp with DropConnect) (Zhao et al., 2021) as well as one ANN. The results of the seven SNNs can be found in Dora et al. (2018), López-Vázquez et al. (2019), and Zhao et al. (2021), while the results for the ANN were obtained by our own experiment using the open-source software library, Keras. For comparison, the ANN we used in this article is a three-layer fully-connected network with a sigmoid activation function, cross-entropy loss, and Adam optimizer. The two STNets used in the experiments were the pseudo-four-layer structures as described in Section 3.3. Since the coding layer in STNet only provides information conversion without performing substantial calculations, the pseudo-four-layer STNet is actually equivalent to a three-layer network in the operating mechanism. The experimental results of the two STNets and the ANN reported for the small datasets were the average of five independent five-fold cross-validation experiments. The reported results on the large datasets were the average of five independent experiments because their training and testing sets have been defined in advance.

### 4.1. Datasets

Eight benchmark datasets including six small datasets, i.e., Sonar, Liver, Ionosphere, Breast cancer, PIMA, Iris, and Wine, and two large datasets, i.e., Statlog Landsat and MNIST, were employed in this article. The MNIST dataset comes from http://yann.lecun.com/exdb/mnist/, and the other seven datasets are from the UCI machine learning repository. Details including the number of features, categories, and samples contained in each dataset are shown in Table 1. It can be seen that there are two-class and multi-class datasets, small and large sample datasets, and few and many feature datasets, so these selected datasets are representative.

**Table 1:** Details of eight benchmark datasets.

**Dataset**	**No. of features**	**No. of categories**	**No. of samples**
Sonar	60	2	208
Liver	6	2	345
Breast cancer	9	2	683
PMIA	8	2	768
Iris	4	3	150
Wine	13	3	178
Statlog Landsat	36	6	6,435
MNIST	28 × 28	10	70,000

During the data preprocessing step, all the datasets only do the min-max normalization except for Breast cancer, MNIST, and Statlog Landsat. As for the Breast cancer dataset, on account of it containing some missing values, we filled them with the average of all existing values in the corresponding feature. For the MNIST dataset, each two-dimensional digital image needs to be pulled into one-dimensional form before being passed into our fully connected networks. The Statlog Landsat dataset contains 36 features, which are composed of four-spectral values of 3 × 3 neighborhood pixels in a satellite image. Since the classification is associated with the central pixel of each neighborhood, we averaged the nine pixels in each band as a new feature as in Zhao et al. (2021). Thus, the Statlog Landsat dataset is reduced from 36 features to four features.

Using dimensionality reduction on a large dataset such as Statlog Landsat can shorten the running time. Therefore, in our experiments, except for the C-STNet which used the original Statlog Landsat with 36 features, all other approaches employ the reduced dimensionality Statlog Landsat with four features. Since the C-STNet does not use the population coding scheme that can ensure the full extraction of enough information from the reduced dimensionality Statlog Landsat through re-upgrading the dataset like other SNNs, it can only operate using the original Statlog Landsat to obtain satisfactory results.

### 4.2. Parameter settings

As is well-known, neural network performance depends not only on weight learning but also on the choice of other key parameters. Therefore, in this subsection, we discuss the selection of some pivotal parameters, including the number of neurons *Q* in the ANN hidden layer, the number of synapses *K*, and the constant β in Equation (6) in the C-STNet, as well as the number of Gaussian fields *G*, the number of SNN neurons *P* and ANN neurons *Q* in the hidden layer in the P-STNet. Here we took the Sonar dataset as an example, and the parameter selection for the other datasets was similar.

**Figure 4:**
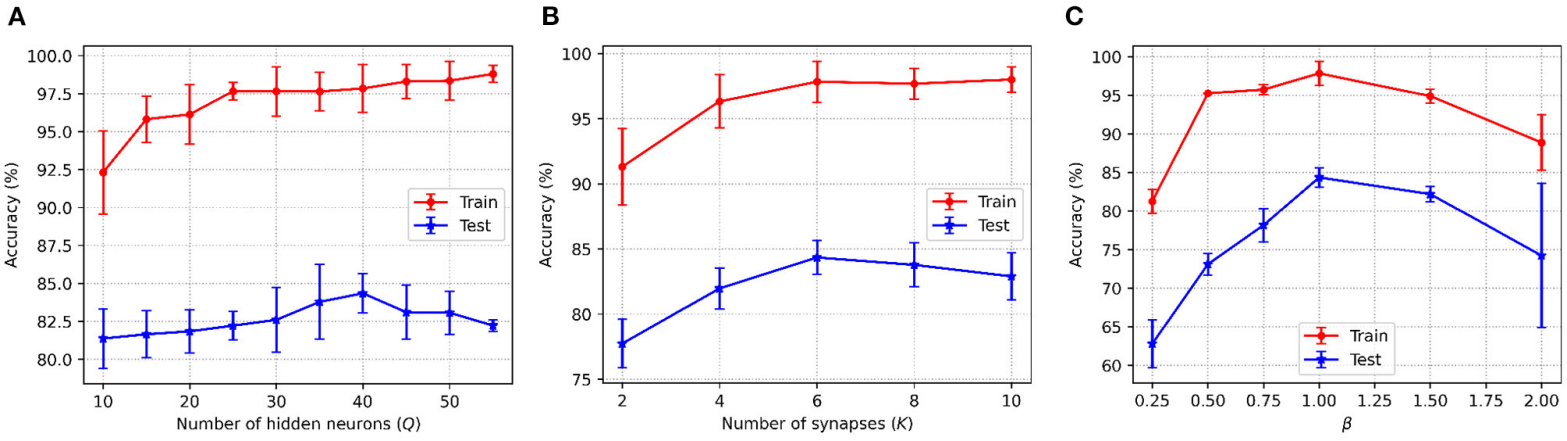
C-STNet accuracy changes with the different values of **(A)**: The number of hidden neurons, **(B)**: The number of synapses, and **(C)**: The constant β in the coding operation.

Figure 4 shows the accuracy changes with the different values of the three key parameters in C-STNet. First, it can be observed from Figure 4A that as *Q* increased from 10 to 50, the training accuracy increases constantly, while the test accuracy goes up first and then goes down. This indicates that more hidden neurons in a certain range can help improve the network performance. Whereas once *Q* is greater than 40, the network is too complex to classify the test dataset well. Therefore, we set *Q* to 40 on the Sonar dataset, and to 200 on the MNIST dataset. The exact values of *Q* used on other datasets can be viewed in Table 3.

Then, Figure 4A, shows that when neurons were connected with six synapses, the C-STNet performed the best. This analysis is the same as that in Figure 4A. Hence, we set *K* to 6 on all the small datasets and 10 on the large Statlog Landsat and MNIST datasets.

Figure 4C exhibits the change of the corresponding accuracy with the constant β in (6) varying from 0.25 to 2. When β is 1, the network accuracy is the highest. If the value of β is too large or too small, it will damage the network performance. Therefore, we set β to 1 for all the datasets.

**Figure 5:**
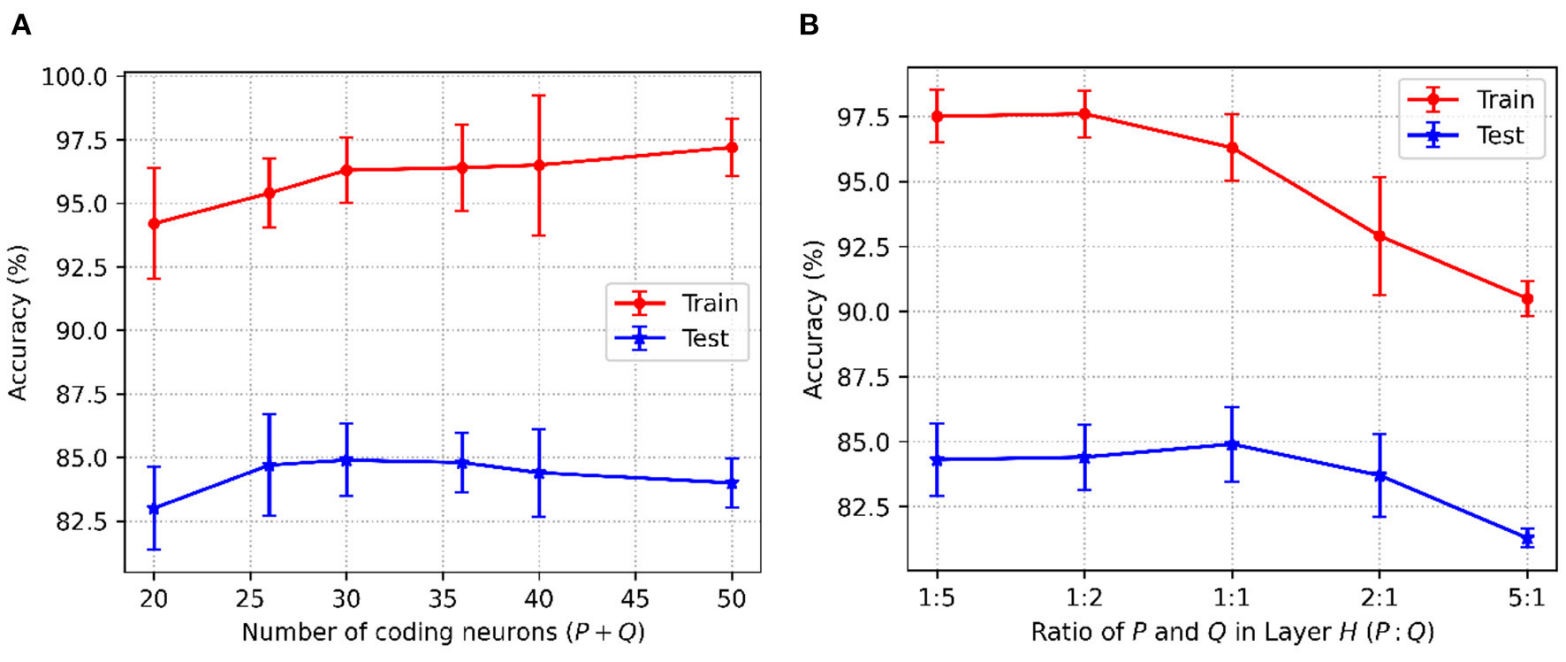
P-STNet accuracy changes with the different values of **(A)**: The number of hidden neurons and **(B)**: The ratio of *P* and *Q* in the hidden layer.

For the P-STNet, the accuracy changes with the number of hidden neurons (*P*+*Q*), and the relationship between *P* and *Q* is shown in Figure 5. Because the Sonar dataset contains more features, linear time delay coding is used here. From Figure 5A, it can be seen that the best result was obtained when the total number of hidden neurons was set to 30. This is because the test accuracy was the highest at this time, and fewer or more hidden neurons will make the network underfit or overfit. Notably, the relationship between *P* and *Q* was not considered here as we had set *P* equal to *Q*. For example, if the number of neurons in the hidden layer *H* was 30, then both *P* and *Q* were 15. Therefore, we set the number of hidden neurons to 30 for the Sonar dataset.

Here we will discuss the influence of the relationship between *P* and *Q* on networkperformance. Assuming that the total number of hidden neurons was a constant of 30, the results of *P* to *Q* ratios set to 1:5, 1:2, 1:1, 2:1 and 5:1 are plotted in Figure 5B. It can be observed that as the ratio decreased from 5 to 0.2, the training accuracy curve showed an upward trend. This indicates that the higher the proportion of the ANN in the P-STNet, the more it helps the network learn better. For the test accuracy, it was the highest when the ratio is 1:1. But once the balance between *P* and *Q* was broken, it was impaired. This manifests that the high proportion of the ANN in the P-STNet can only unilaterally improve the training performance, but damages the generalization ability of the network. On the contrary, a larger proportion of the SNN causes a decrease in classification accuracy, but it guarantees a certain generalization ability. According to the above discussion, the balance between *P* and *Q* can cause the P-STNet to exhibit both good classification ability and generalization ability. Therefore, we set *P* equal to *Q* for the Sonar dataset and set the ratios of *P* to *Q* to approximately 1 for the other datasets. The specific values of *P* and *Q* can be referred to in Table 3, which are set to 150 on the MNIST dataset.

In addition, the number of Gaussian fields *G* in the population coding and the learning rate are also very important parameters. Their selection schemes are the same as the above and only the results of parameter selection are given. For the number of Gaussian fields used in the P-STNet, it was finally set to 5 for the Liver dataset, and to 6 for the other datasets except for Sonar and MNIST. For the learning rates of the two STNets, the down-regulated learning rate with a decay rate of 0.99 and 0.95 as well as an initial value of 0.1 was used for the Statlog Landsat and PIMA datasets, respectively, and a fixed learning rate of 0.03 was employed for other datasets. The empirical settings of some extra parameters are shown in Table 2.

**Table 2:** Empirical settings of some parameters in all experiments.

**Parameter**	**Description**	**Value**
τ	Time constant of the spike response kernel	7
*T*	Spiking time window limit	10
*d* ^*k*^	Every time delay	*k*
θ	Potential threshold	1

### 4.3. Experimental results

In order to evaluate the performance of C-STNet and P-STNet, the experimental results for six small datasets (i.e., Sonar, Liver, Breast cancer, PIMA, Iris, and Wine) are compared with other five SNNs, i.e., SpikeProp, SWAT, SRESN, TMM-SNN, and GE-SNN as well as an ANN. Table 3 summarizes the detailed results involving the network architecture, the number of epochs, training accuracy, and testing accuracy, where the architecture column exhibits the number of neurons in each layer of the corresponding approaches. For the C-STNet and P-STNet, they are in the form of (*N*+1):*Q*:*J* and (*N*×*G*+(*N*+1)):(*P*+*Q*):*J* or (*N*+(*N*+1)):(*P*+*Q*):*J*, respectively. The bold numbers in the table indicate the best results of the corresponding evaluation indicators among all approaches.

**Table 3:** Experimental results of eight approaches on the six little datasets.

**Dataset**	**Approach**	**Architecture**	**# epochs**	**Training accuracy (%)**	**Testing accuracy (%)**	**Rank**
Sonar	SpikeProp	60:30:02	88	84.7 (1.6)	80.1 (3.9)	4
	ANN	60:40:02	100	95.8 (0.5)	84.6 (2.4)	2
	C-STNet	61:40:02	**40**	**97.8 (1.6)**	84.3 (1.3)	3
	P-STNet	(60 + 61):(15 + 15):2	52	96.3 (1.3)	**84.9 (1.4)**	**1**
Liver	SpikeProp	37:15:02	3000	71.5 (5.2)	65.1 (4.7)	6
	SWAT	36:468:2	500	**74.8 (2.1)**	60.9 (3.2)	7
	SRESN	36:(6–9)	715	60.4 (1.7)	59.7 (1.7)	8
	TMM-SNN	35:(5–8):2	442	74.2 (3.5)	70.4 (2.0)	3
	GE-SNN	-	-	76.4 (2.0)	67.2 (3.0)	5
	ANN	6:15:02	100	74.6 (1.5)	71.8 (0.7)	2
	C-STNet	7:15:02	68	69.4 (2.8)	69.0 (1.2)	4
	P-STNet	(30 + 7): (7 + 7):2	**57**	72.3 (1.3)	**72.0 (0.9)**	**1**
Breast cancer	SpikeProp	55:15:02	1000	97.3 (0.6)	97.2 (0.6)	2
	SWAT	54:702:2	500	96.5 (0.5)	95.8 (1.0)	8
	SRESN	54: (8–12)	306	**97.7 (0.6)**	97.2 (0.7)	2
	TMM-SNN	54: (2–8):2	70	97.4 (0.3)	97.2 (0.5)	2
	GE-SNN	-	-	97.8 (0.7)	95.9 (0.8)	7
	ANN	9:15:02	100	97.5 (0.1)	97.1 (0.2)	5
	C-STNet	10:14:02	**53**	97.4 (0.5)	97.1 (0.2)	5
	P-STNet	(54 + 10): (8 + 7):2	39	97.4 (0.1)	**97.3 (0.1)**	**1**
PIMA	SpikeProp	49:20:02	3000	78.6 (2.5)	76.2 (1.8)	5
	SWAT	54:702:2	500	77.0 (2.1)	72.1 (1.8)	7
	SRESN	48: (9–14)	254	70.5 (2.4)	69.9 (2.1)	8
	TMM-SNN	48: (5–14):2	160	**79.7 (2.3)**	78.1 (1.7)	2
	GE-SNN	-	-	79.0 (1.3)	74.8 (1.3)	6
	ANN	8:15:02	100	78.4 (0.2)	77.4 (0.2)	3
	C-STNet	9:15:02	**50**	78.2 (0.2)	**78.2 (0.5)**	**1**
	P-STNet	(48 + 9): (8 + 7):2	59	78.5 (0.3)	77.4 (0.4)	3
Iris	SpikeProp	25:10:03	1000	97.2 (1.9)	96.7 (1.6)	6
	SWAT	204–312–3	500	96.7 (1.4)	92.4 (1.7)	8
	SRESN	24: (6–10)	102	96.9 (1.0)	97.3 (1.3)	3
	TMM-SNN	24: (4–7):3	94	97.5 (0.8)	97.2 (1.0)	4
	GE-SNN	-	-	**99.2 (0.7)**	93.9 (2.1)	7
	ANN	4:10:03	100	97.7 (0.3)	97.1 (0.3)	5
	C-STNet	5:10:03	42	97.7 (0.2)	**97.7 (0.5)**	**1**
	P-STNet	(24 + 5): (5 + 5):3	**37**	98.0 (0.3)	97.6 (0.5)	2
Wine	SpikeProp	79:10:03	1000	99.2 (1.2)	96.8 (1.6)	5
	SWAT	78:1014:3	500	98.6 (1.1)	92.3 (2.4)	6
	SRESN	78: (5–10)	128	96.9 (1.6)	91.0 (1.2)	7
	TMM-SNN	78:03:03	80	**100 (0)**	97.5 (0.8)	4
	GE-SNN	-	-	96.4 (1.6)	86.8 (4.6)	8
	ANN	13:08:03	100	99.9 (0.1)	**99.1 (0.3)**	**1**
	C-STNet	14:08:03	**41**	99.9 (0.2)	98.8 (0.4)	3
	P-STNet	(78 + 14): (4 + 4):3	57	99.9 (0.2)	**99.1 (0.6)**	**1**

The bold numbers indicate the best results of the corresponding evaluation indicators among all approaches.

From a rough comparison in Table 3, it can be seen that the number of epochs required by the two STNets is relatively small. For the Sonar dataset with many features, it can be seen that the training accuracy and test accuracy of two STNets and ANN were significantly higher than those of SpikeProp. This indicates that the addition of ANN operations helps the neural network to classify multi-feature datasets. Although the test accuracy of the C-STNet was lower than the ANN, both the training and test accuracies of the P-STNet are higher than the ANN. This suggests that the structure that processes continuous spatial information and discrete temporal information in parallel is better than the serial structure or the ANN for multi-feature classification tasks. On the other five datasets with fewer features, although the training accuracies of the two STNets were generally lower than some other approaches, the best results of the testing accuracy were always from one of these two STNets. This implies that the two STNets have good generalization performance. To visually compare the test accuracies of all approaches, we ranked the test results for each dataset, as listed in the last column of Table 3. Due to incomplete approaches for comparison for the Sonar dataset, we averaged this ranking from the other five datasets, and the results are displayed in Table 4. It can be clearly observed that the P-STNet ranked the highest, followed by the C-STNet. This demonstrates that the test accuracies of the two STNets were generally better than the other SNNs and even the ANN.

**Table 4:** Average rankings of the test accuracies for the eight approaches on the six small datasets.

**SpikeProp**	**SWAT**	**SRESN**	**TMM-SNN**	**GE-SNN**	**ANN**	**C-STNet**	**P-STNet**
4.8	7.2	5.6	3.0	6.6	3.2	**2.8**	**1.6**

The bold values indicate the top two best results among all comparing approaches.

The results of test accuracy were also supported by the Friedman test (Wang et al., 2020) with a confidence level of 0.05. The *p*-value of 3.1275e-05 indicates that there was a statistically significant difference between all approaches. The Nemenyi post hoc test (Wang et al., 2020) was executed in pairs for any two approaches. Only the Nemenyi post hoc test *p*-value for the pairwise comparison between the P-STNet and SWAT of 0.0115 was lower than 0.05, which demonstrated that the P-STNet outperformed SWAT with a statistical significance at the 95% confidence level. Judging from the *p*-values between SWAT and other approaches given in Table 5, the P-STNet also obtained the best testing accuracy in terms of statistical significance among all approaches, followed by the C-STNet.

**Table 5:** Nemenyi post hoc test *p*-values for pairwise comparison between SWAT and other seven approaches.

	**SpikeProp**	**SRESN**	**TMM-SNN**	**GE-SNN**	**ANN**	**C-STNet**	**P-STNet**
SWAT	0.8304	0.9000	0.1624	0.9000	0.2467	**0.1011**	**0.0115**

The bold values indicate the top two best results among all comparing approaches.

**Figure 6:**
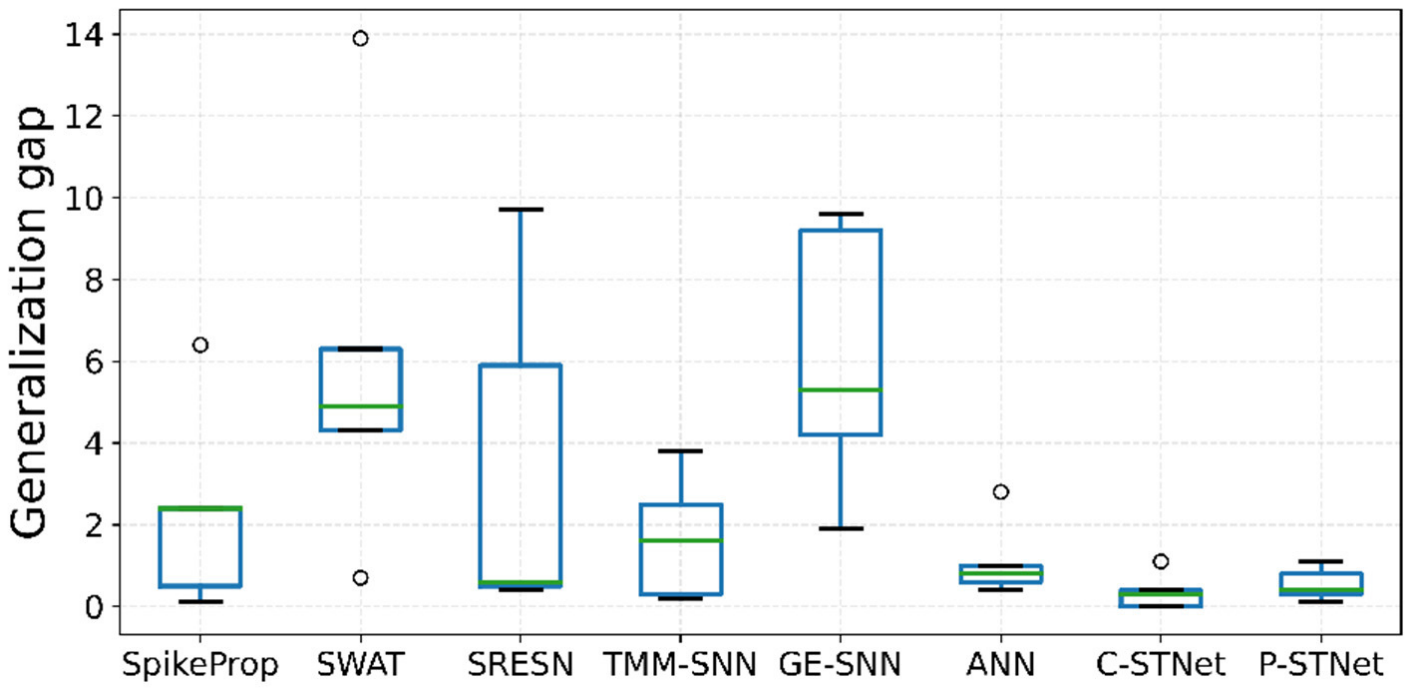
Boxplots of the generalization gaps on the five small datasets for the eight approaches.

Regarding the evaluation of the generalization of the C-STNet and P-STNet, Figure 6 exhibits the boxplots of the generalization gaps for the eight approaches for the five small datasets excluding Sonar. The generalization gap was defined as the difference between training and testing accuracy (Hoffer et al., 2017), which can be easily calculated from Table 3. The boxplot corresponding to each approach was drawn according to the minimum, first quartile, median, third quartile, and maximum values obtained by the generalization gaps for the five small datasets. It can be seen that although there was an outlier (caused by the large generalization gap for the Wine dataset) existing in the C-STNet boxplot, the distances between the maximum and minimum of the two-STNet boxplots were much shorter than those of other approaches, indicating that the generalization gap values of the two STNets for the five datasets were more concentrated. Moreover, the minimum, first quartile, median, third quartile, and maximum values of the boxplots for the two STNets were all lower than or equal to the corresponding parts of the other approach boxplots, which showed that the C-STNet and the P-STNet have better generalization abilities.

**Figure 7:**
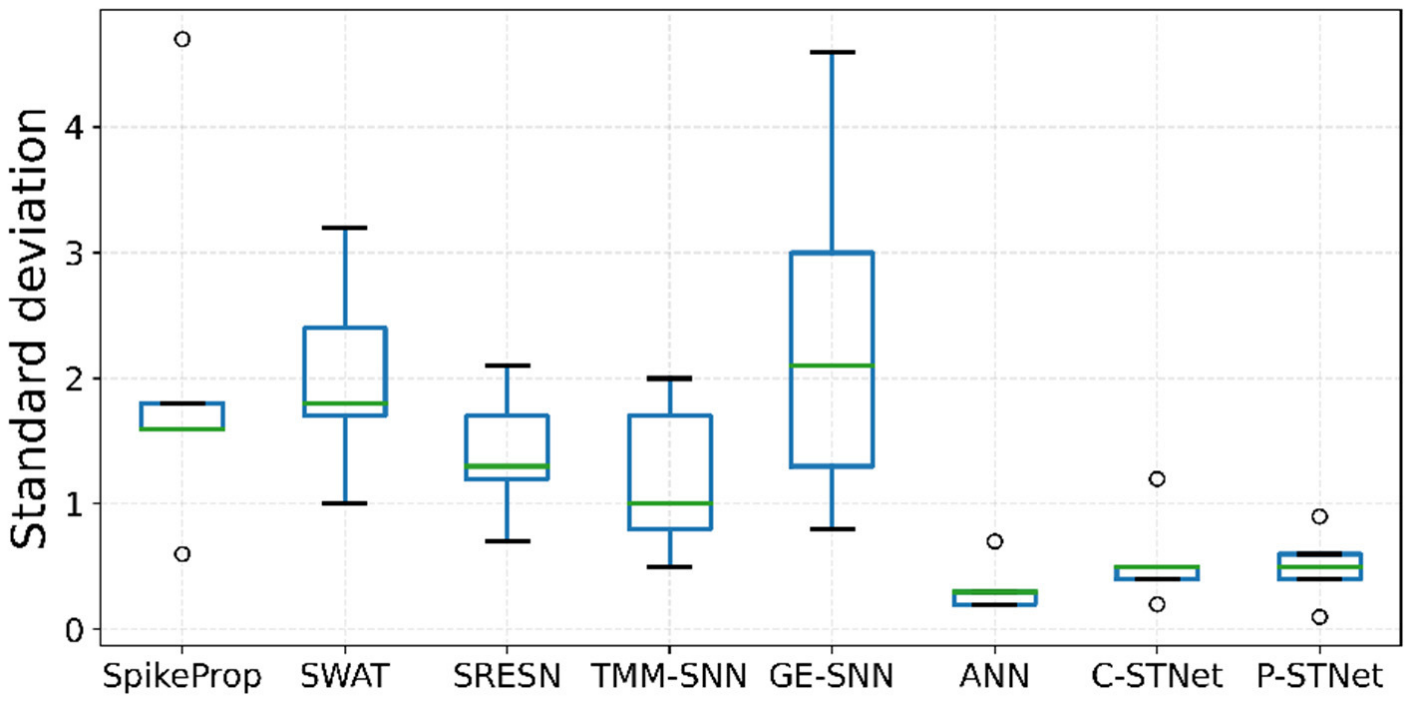
Boxplots of the test-accuracy standard deviations on the five small datasets for the eight approaches.

In order to evaluate the stabilities of C-STNet and P-STNet, the boxplots of the test-accuracy standard deviations for the eight approaches on the five small datasets except for Sonar are plotted in Figure 7. It can be observed that the minimum, first quartile, median, third quartile, and maximum values as well as outliers of the boxplots of the ANN and the two STNets were much lower than those of other approaches. Besides, the range of lines from the maximum outlier to the minimum value of the ANN and the ranges of lines from the maximum outlier to the minimum outlier of the two STNets were relatively short. These illustrate that the test-accuracy standard deviations of the ANN and the two STNets were usually lower than other approaches, and did not rely on the datasets. This further demonstrated that the stabilities of multiple independent experimental results of the ANN and the two STNets were better than the others.

**Figure 8:**
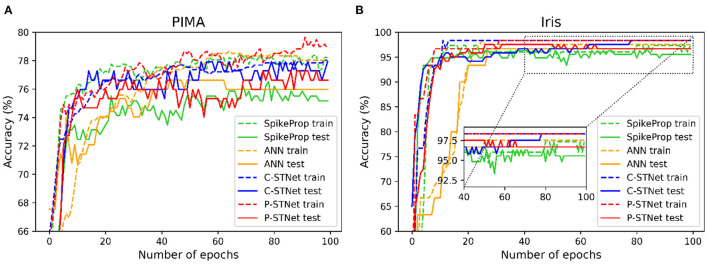
Accuracy curves change as the number of epochs for four approaches on **(A)**: The two-classified PIMA dataset and **(B)**: The multi-classified Iris dataset.

In order to evaluate the convergence of C-STNet and P-STNet, we took a two-category PIMA dataset and a multi-category Iris dataset as examples, and plotted the accuracy curves of four approaches (SpikeProp, ANN, C-STNet, and P-STNet) as the number of epochs on these two datasets, as shown in Figure 8. From the results for these two datasets, we can see that all accuracy curves as the epoch first increased quickly, then increased slowly, and finally stabilized. However, it is clear that the convergence speeds of the three SNNs were faster than that of the ANN. The accuracy results of the ANN and the two STNets that could be converged were higher than those of SpikeProp. In brief, the C-STNet and the P-STNet performed well in terms of convergence speed and accuracy.

**Figure 9:**
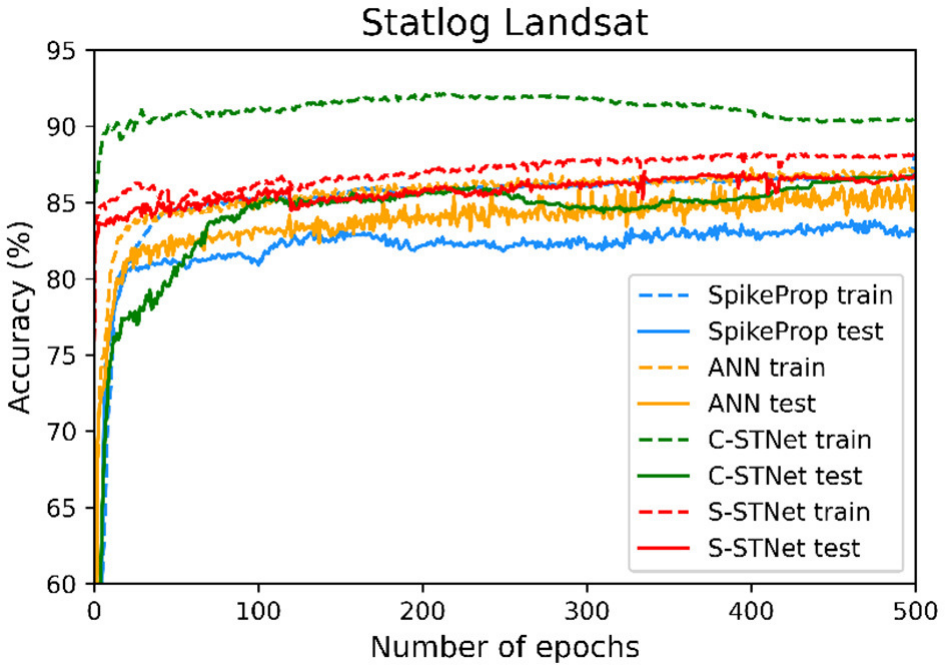
Accuracy curves of the four approaches varying with the number of epochs.

In order to access the classification ability of the C-STNet and the P-STNet on larger datasets, we conducted experiments using the Statlog Landsat dataset to compare the two STNets with four other approaches (i.e., SpikeProp, SPDO, SPDC, and an ANN). From the experimental results as shown in Table 6, it can be seen that the two STNets acquired the best testing accuracies. The accuracy curves of the four approaches varying with the number of epochs in Figure 9, shows that the two STNets achieved the best testing accuracies. In addition, the training accuracy of C-STNet was significantly higher than other approaches. This may be because, unlike the other methods, the C-STNet used the original Statlog Landsat dataset with 36 features, which may have enabled the C-STNet to obtain richer information from the dataset to produce a high training accuracy.

**Table 6:** Results of six approaches on the Statlog Landsat dataset.

**Approach**	**Architecture**	**# epochs**	**Training accuracy (%)**	**Testing accuracy (%)**
SpikeProp	101:70:6	500	85.8 (0.16)	80.3 (0.18)
SPDO	101:70:6	500	88.4 (0.08)	85.3 (0.06)
SPDC	101:70:6	500	88.9 (0.08)	85.9 (0.07)
ANN	4:25:6	500	86.7 (0.47)	86.1 (0.35)
C-STNet	37:25:6	303	**91.5 (0.75)**	**86.4 (0.49)**
P-STNet	(24 + 5):(12 + 12):6	**220**	87.6 (0.44)	**86.4 (0.79)**

The bold values indicate the best result of the corresponding evaluation indicators among all comparing approaches.

**Figure 10:**
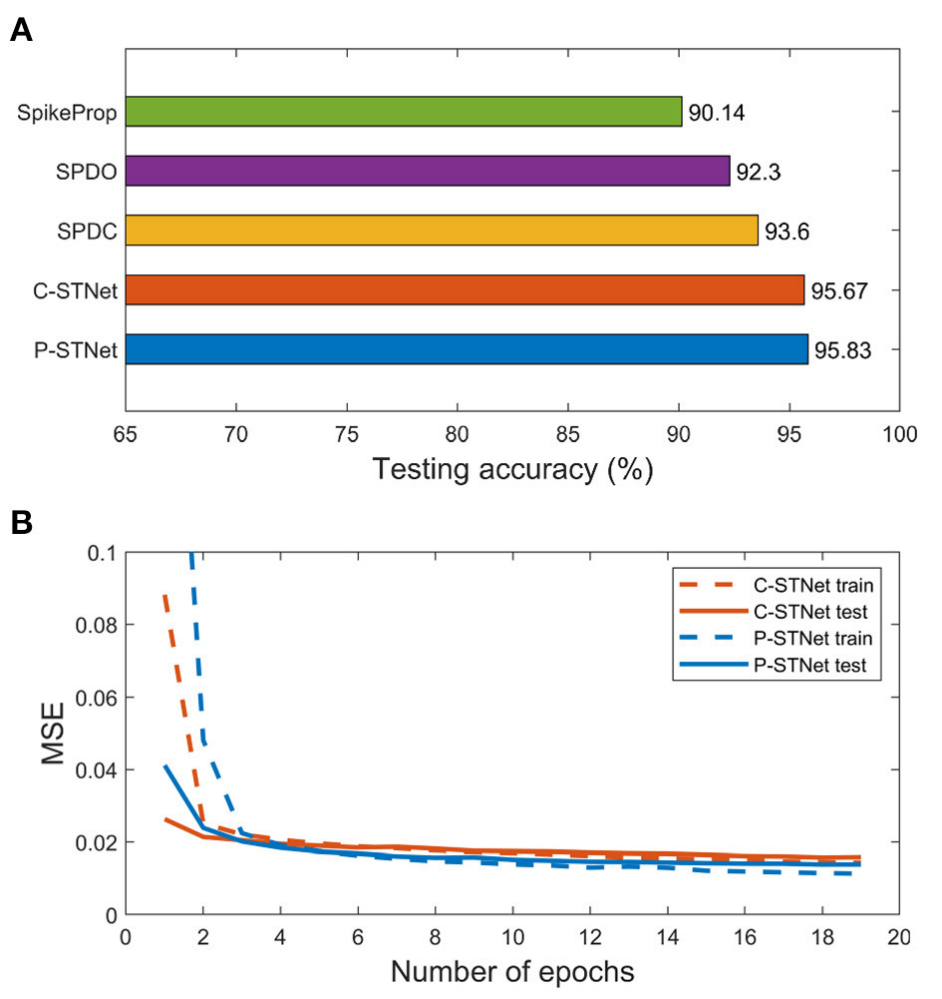
Experimental results on the MNIST dataset. **(A)**: Testing accuracies of the five approaches. **(B)**: MSE curves of the two STNets.

We further conducted experiments on a larger-scale image dataset, MNIST. Here the STNet structure used was a three-layer fully connected perceptron. Since the MNIST dataset contains many features, linear time delay coding was used for the P-STNet. The experimental results of the two STNets are given in Figure 10. The testing accuracies compared with SpikeProp, SPDO, and SPDC are plotted in Figure 10A, where the result of SpikeProp is from Arora et al. (2019) and the results of both the STNets are the average from three experimental runs. It can be clearly seen that our two STNets performed much better than the other three SNN-only approaches due to the addition of the ANN components, suggesting that the combination of an ANN and an SNN can indeed improve the performance of an SNN alone.

Although the classification results of the two STNets on the MNIST dataset were worse than convolutional neural networks, they were better than networks that used SpikeProp alone. This may be due to the fact that both the SNN and the ANN in STNet are not good at processing image datasets. Nonetheless, the experimental results were enough to prove that the idea of combining an ANN and an SNN is feasible and can greatly improve the SNN performance, which motivates us to combine more advanced SNN components with an ANN in the future.

Figure 10B shows the MSE curves of two STNets on the number of epochs. It can be seen that starting from the 3rd epoch, the gaps between the training and testing MSE curves of the two STNets became very small, and remained small as the epoch increased. This indicates that experiments on large datasets can indeed avoid overfitting, and further shows that our approaches can enhance the generalization ability by extracting two kinds of information.

In summary, the P-STNet was better than C-STNet for the Sonar, Liver, Breast cancer, Wine, and MNIST datasets in terms of test accuracy, while it was not as good as the C-STNet for the PIMA and Iris datasets. Their results were the same on the Statlog Landsat dataset. According to the comprehensive ranking and statistical comparison discussed above, the P-STNet was a little better than the C-STNet on the whole. The reason for these results may be that the parallel mechanism of the P-STNet can extract both temporal and spatial information of datasets from shallow layers to deep layers, which focuses on mining the breadth of information, while the serial mechanism of the C-STNet focuses on mining the depth of information. However, the series and parallel combinations may produce different results in different application scenarios. The specific choice of either the C-STNet or the P-STNet should depend on the specific tasks and datasets.

## 5. Conclusion

In view of the cognitive manner that the visual cortex processes visual information, this article proposes two types of Spatio-Temporal Combined Network (STNet): a concatenated version, C-STNet, and a parallel version, P-STNet. The C-STNet is a front and back splicing form of an ANN and an SNN, which is completed by converting continuous signals into discrete spiking time series. The P-STNet is composed of ANN and SNN calculations in parallel followed by an SNN, which realizes the simultaneous extraction and processing of spatio-temporal information. Finally, to evaluate the performance of the C-STNet and the P-STNet, experiments were conducted on six small and two large classification datasets. The comparison results among the two STNets and eight other popular approaches showed that the two STNets performed better in terms of testing accuracy, generalization, stability, and convergence. Furthermore, the P-STNet had higher testing accuracy than the C-STNet on the whole. These promising results warrant future investigations which may continue to simulate neurobiological research findings to design a more brain-like SNN to further improve its performance.

## Data availability statement

The original contributions presented in the study are included in the article/supplementary material, further inquiries can be directed to the corresponding author.

## Author contributions

JY, FL, and WT conceived the study. FL and WT conducted the experiment(s) and analyzed the results. FL and JY wrote and reviewed the paper. JY, WW, and JW supervised. All authors contributed to the article and approved the submitted version.

## References

[B1] Abiodun O. I. Jantan A. Omolara A. E. Dada K. V. Umar A. M. Linus O. U.. (2019). Comprehensive review of artificial neural network applications to pattern recognition. IEEE Access 7, 158820–158846. 10.1109/ACCESS.2019.2945545

[B2] Amin H. H. Deabes W. Bouazza K. (2017). “Clustering of user activities based on adaptive threshold spiking neural networks,” in 2017 Ninth International Conference on Ubiquitous and Future Networks (ICUFN) (Milan: IEEE), 1–6. 10.1109/ICUFN.2017.7993735

[B3] Arora T. Vatsa M. Singh R. (2019). Synaptic Weight Update in Deep Spiking Neural Networks. Available online at: https://repository.iiitd.edu.in/xmlui/bitstream/handle/123456789/775/2015107_TUSHAR%20ARORA.pdf?equence=1&isAllowed=y

[B4] Bohte S. M. Kok J. N. La Poutre H. (2002). Error-backpropagation in temporally encoded networks of spiking neurons. Neurocomputing 48, 17–37. 10.1016/S0925-2312(01)00658-0

[B5] Cheng X. Zhang T. Jia S. Xu B. (2020). Finite meta-dynamic neurons in spiking neural networks for spatio-temporal learning. arXiv preprint arXiv:2010.03140. 10.48550/arXiv.2010.03140

[B6] Davidson S. Furber S. B. (2021). Comparison of artificial and spiking neural networks on digital hardware. Front. Neurosci. 15, 651141. 10.3389/fnins.2021.651141 33889071 PMC8055931

[B7] Dora S. Subramanian K. Suresh S. Sundararajan N. (2016). Development of a self-regulating evolving spiking neural network for classification problem. Neurocomputing 171, 1216–1229. 10.1016/j.neucom.2015.07.086

[B8] Dora S. Sundaram S. Sundararajan N. (2018). An interclass margin maximization learning algorithm for evolving spiking neural network. IEEE Trans. Cybern. 49, 989–999. 10.1109/TCYB.2018.2791282 29994611

[B9] Fu Q. Dong H. (2022). Spiking neural network based on multi-scale saliency fusion for breast cancer detection. Entropy 24, 1543. 10.3390/e24111543 36359633 PMC9689387

[B10] Hao Y. Huang X. Dong M. Xu B. (2020). A biologically plausible supervised learning method for spiking neural networks using the symmetric stdp rule. Neural Networks 121, 387–395. 10.1016/j.neunet.2019.09.007 31593843

[B11] He Y. Nie S. Liu R. Jiang S. Shi Y. Wan Q. (2019). Spatiotemporal information processing emulated by multiterminal neuro-transistor networks. Adv. Mater. 31, 1900903. 10.1002/adma.201900903 30957923

[B12] Hoffer E. Hubara I. Soudry D. (2017). “Train longer, generalize better: closing the generalization gap in large batch training of neural networks,” in Advances in Neural Information Processing Systems, Vol. 30. 10.48550/arXiv.1705.08741

[B13] Huang Y. Xu J. Zhou Y. Tong T. Zhuang X. ADNI. (2019). Diagnosis of Alzheimer's disease via multi-modality 3d convolutional neural network. Front. Neurosci. 13, 509. 10.3389/fnins.2019.00509 31213967 PMC6555226

[B14] Joukal M. (2017). “Anatomy of the human visual pathway,” 19 in Homonymous Visual Field Defects, 1–16. 10.1007/978-3-319-52284-5_1

[B15] Kang T. Ding W. Zhang L. Ziemek D. Zarringhalam K. (2017). A biological network-based regularized artificial neural network model for robust phenotype prediction from gene expression data. BMC Bioinform. 18, 1–11. 10.1186/s12859-017-1984-2 29258445 PMC5735940

[B16] Kasabov N. K. (2014). Neucube: a spiking neural network architecture for mapping, learning and understanding of spatio-temporal brain data. Neural Networks 52, 62–76. 10.1016/j.neunet.2014.01.006 24508754

[B17] Kheradpisheh S. R. Ganjtabesh M. Thorpe S. J. Masquelier T. (2018). Stdp-based spiking deep convolutional neural networks for object recognition. Neural Networks 99, 56–67. 10.1016/j.neunet.2017.12.005 29328958

[B18] Kheradpisheh S. R. Masquelier T. (2020). Temporal backpropagation for spiking neural networks with one spike per neuron. Int. J. Neural Syst. 30, 2050027. 10.1142/S0129065720500276 32466691

[B19] Li Y. Ma W. (2010). “Applications of artificial neural networks in financial economics: a survey,” in 2010 International Symposium on Computational Intelligence and Design, volume 1 (Hangzhou: IEEE), 211–214.

[B20] López-Vázquez G. Ornelas-Rodriguez M. Espinal A. Soria-Alcaraz J. A. Rojas-Domínguez A. Puga-Soberanes H.. (2019). Evolutionary spiking neural networks for solving supervised classification problems. Comput. Intell. Neurosci. 2019, 4182639. 10.1155/2019/4182639 31049050 PMC6458934

[B21] Muramatsu N. Yu H.-T. (2021). Combining spiking neural network and artificial neural network for enhanced image classification. arXiv preprint arXiv:2102.10592. 10.48550/arXiv.2102.10592

[B22] Nguyen D.-A. Tran X.-T. Iacopi F. (2021). A review of algorithms and hardware implementations for spiking neural networks. J. Low Power Electron. Appl. 11, 23. 10.3390/jlpea11020023

[B23] Nobukawa S. Nishimura H. Yamanishi T. (2019). Pattern classification by spiking neural networks combining self-organized and reward-related spike-timing-dependent plasticity. J. Artif. Intell. Soft Comput. Res. 9, 283–291. 10.2478/jaiscr-2019-0009

[B24] Pan Z. Wu J. Zhang M. Li H. Chua Y. (2019). “Neural population coding for effective temporal classification,” in 2019 International Joint Conference on Neural Networks (IJCNN) (Budapest: IEEE), 1–8.

[B25] Rafi T. H. (2021). A brief review on spiking neural network-a biological inspiration. Preprints 2021, 2021040202. 10.20944/preprints202104.0202.v132283112

[B26] Rueckauer B. Lungu I.-A. Hu Y. Pfeiffer M. Liu S.-C. (2017). Conversion of continuous-valued deep networks to efficient event-driven networks for image classification. Front. Neurosci. 11, 682. 10.3389/fnins.2017.00682 29375284 PMC5770641

[B27] Seo K.-K. (2013). A simulation study on an artificial neural network based automatic control system of a plant factory. Int. J. Control Automat. 6, 127–136. 10.14257/ijca.2013.6.5.12

[B28] Stewart K. M. Neftci E. O. (2022). Meta-learning spiking neural networks with surrogate gradient descent. Neuromorphic Comput. Eng. 2, 044002. 10.1088/2634-4386/ac8828

[B29] Taherkhani A. Belatreche A. Li Y. Cosma G. Maguire L. P. McGinnity T. M. (2020). A review of learning in biologically plausible spiking neural networks. Neural Networks 122, 253–272. 10.1016/j.neunet.2019.09.036 31726331

[B30] Wade J. J. McDaid L. J. Santos J. A. Sayers H. M. (2010). Swat: a spiking neural network training algorithm for classification problems. IEEE Trans. Neural Networks 21, 1817–1830. 10.1109/TNN.2010.2074212 20876015

[B31] Wang K. Cao J. Pei H. (2020). Robust extreme learning machine in the presence of outliers by iterative reweighted algorithm. Appl. Math. Comput. 377, 125186. 10.1016/j.amc.2020.125186

[B32] Wang W. Pedretti G. Milo V. Carboni R. Calderoni A. Ramaswamy N.. (2018). Learning of spatiotemporal patterns in a spiking neural network with resistive switching synapses. Sci. Adv. 4, eaat4752. 10.1126/sciadv.aat4752 30214936 PMC6135543

[B33] Xie X. Wen S. Yan Z. Huang T. Chen Y. (2020). Designing pulse-coupled neural networks with spike-synchronization-dependent plasticity rule: image segmentation and memristor circuit application. Neural Comput. Appl. 32, 13441–13452. 10.1007/s00521-020-04752-7

[B34] Xu Q. Qi Y. Yu H. Shen J. Tang H. Pan G.. (2018). “Csnn: an augmented spiking based framework with perceptron-inception,” in IJCAI, 1646–1652. 10.24963/ijcai.2018/228

[B35] Zhang M. Wang J. Wu J. Belatreche A. Amornpaisannon B. Zhang Z.. (2021). Rectified linear postsynaptic potential function for backpropagation in deep spiking neural networks. IEEE Trans. Neural Networks Learn. Syst. 33, 1947–1958. 10.1109/TNNLS.2021.3110991 34534091

[B36] Zhao J. Yang J. Wang J. Wu W. (2021). Spiking neural network regularization with fixed and adaptive drop-keep probabilities. IEEE Trans. Neural Networks Learn. Syst. 33, 4096–4109. 10.1109/TNNLS.2021.3055825 33571100

